# Right ventricular wall motion abnormalities n Thalassemia Major patients

**DOI:** 10.1186/1532-429X-16-S1-P257

**Published:** 2014-01-16

**Authors:** Antonella Meloni, Vincenzo Positano, Sabrina Armari, Giuseppina Secchi, Giancarlo Izzi, Cristina Salvatori, Claudio Ascioti, Gennaro Restaino, Massimo Lombardi, Alessia Pepe

**Affiliations:** 1 CMR Unit, Fondazione G.Monasterio CNR-Regione Toscana and Institute of Clinical Physiology, Pisa, Italy; 2 Reparto di Pediatria, Azienda Ospedaliera di Legnago U.O., Legnago, Italy; 3 Servizio trasfusionale, Azienda USL n° 1, Sassari, Italy; 4 Dipartimento Materno-Infantile U.O. Pediatria Oncoematologia, Azienda Ospedaliera di Parma, Parma, Italy; 5 Struttura Complessa di Cardioradiologia, P.O. "Giovanni Paolo II", Lamezia Terme, Italy; 6 Dipartimento di Radiologia, Università Cattolica del Sacro Cuore - Centro di Ricerca e Formazione ad Alta Tecnologia nelle Scienze Biomediche, Campobasso, Italy

## Background

Movement abnormalities in the left ventricle (LV) were shown to be not really frequent in thalassemia major (TM) patients but they were associated with age, myocardial iron overload, LV dilation and dysfunction, and myocardial fibrosis. No data are available about the prevalence and the correlates of right ventricular (RV) wall motion abnormalities. This study investigated the relationship between RV and LV motion abnormalities and between RV motion and function in a large cohort of well-treated TM patients.

## Methods

CMR was performed in 1092 TM patients (537 male; 30.6 ± 8.5 years) enrolled in the Myocardial Iron Overload in Thalassemia (MIOT) Network. Cine images were acquired to evaluate wall motion and to quantify RV volumes and ejection fraction (EF).

## Results

Abnormal motion of the LV was found in 66 (6%) patients (60 hypokinetic and 6 dyskinetic). Abnormal motion of the RV was found in 35 (3.2%) patients (29 hypokinetic, 5 dyskinetic and 1 akynetic). Abnormal LV motion was not correlated with abnormal RV motion. Seventeen patients showed movement abnormalities in both ventricles. The Table 1 shows the comparison between TM patients with normal and abnormal RV motion. Patients with abnormal RV motion were older and they were more frequently males. Right volumes indexed by body surface area (BSA) were significantly higher in patients with abnormal RV motion while the EF was significantly lower.

**Table 1:** 

	Abnormal RV motion	Normal RV motion	P
*Age*	33.9 ± 5.9	30.5 ± 8.6	0.013

*Sex (M/F)*	27/8	510/547	0.001

*RV end-diastolic volume index (ml/m2)*	110.4 ± 48.2	83.4 ± 19.2	< 0.0001

*RV end-systolic volume index (ml/m2)*	61.5 ± 29.6	32.5 ± 11.4	< 0.0001

*RV ejection fraction (%)*	44.9 ± 10.1	61.4 ± 7.7	< 0.0001

## Conclusions

In TM patients movement abnormalities in the right ventricle were less frequent compared to the left ventricle, but were associated with age, sex, RV dilation and dysfunction.

## Funding

The MIOT project receives "no-profit support" from industrial sponsorships (Chiesi Farmaceutici S.p.A. and ApoPharma Inc.). This study was also supported by: "Ministero della Salute, fondi ex art. 12 D.Lgs. 502/92 e s.m.i., ricerca sanitaria finalizzata anno 2006" and "Fondazione L. Giambrone".

